# Cholinergic Modulation of Glial Function During Aging and Chronic Neuroinflammation

**DOI:** 10.3389/fncel.2020.577912

**Published:** 2020-10-15

**Authors:** Rashmi Gamage, Ingrid Wagnon, Ilaria Rossetti, Ryan Childs, Garry Niedermayer, Rose Chesworth, Erika Gyengesi

**Affiliations:** ^1^Department of Pharmacology, School of Medicine, Western Sydney University, Penrith, NSW, Australia; ^2^School of Science, Western Sydney University, Penrith, NSW, Australia; ^3^School of Medicine, Western Sydney University, Penrith, NSW, Australia

**Keywords:** cholinergic system, microglia, astrocytes, basal forebrain, neuroinflammation, aging, neurodegeneration

## Abstract

Aging is a complex biological process that increases the risk of age-related cognitive degenerative diseases such as dementia, including Alzheimer’s disease (AD), Lewy Body Dementia (LBD), and mild cognitive impairment (MCI). Even non-pathological aging of the brain can involve chronic oxidative and inflammatory stress, which disrupts the communication and balance between the brain and the immune system. There has been an increasingly strong connection found between chronic neuroinflammation and impaired memory, especially in AD. While microglia and astrocytes, the resident immune cells of the central nervous system (CNS), exerting beneficial effects during the acute inflammatory phase, during chronic neuroinflammation they can become more detrimental. Central cholinergic circuits are involved in maintaining normal cognitive function and regulating signaling within the entire cerebral cortex. While neuronal-glial cholinergic signaling is anti-inflammatory and anti-oxidative, central cholinergic neuronal degeneration is implicated in impaired learning, memory sleep regulation, and attention. Although there is evidence of cholinergic involvement in memory, fewer studies have linked the cholinergic anti-inflammatory and anti-oxidant pathways to memory processes during development, normal aging, and disease states. This review will summarize the current knowledge of cholinergic effects on microglia and astroglia, and their role in both anti-inflammatory and anti-oxidant mechanisms, concerning normal aging and chronic neuroinflammation. We provided details on how stimulation of α7 nicotinic acetylcholine (α7nACh) receptors can be neuroprotective by increasing amyloid-β phagocytosis, decreasing inflammation and reducing oxidative stress by promoting the nuclear factor erythroid 2-related factor 2 (Nrf2) pathways and decreasing the release of pro-inflammatory cytokines. There is also evidence for astroglial α7nACh receptor stimulation mediating anti-inflammatory and antioxidant effects by inhibiting the nuclear factor-κB (NF-κB) pathway and activating the Nrf2 pathway respectively. We conclude that targeting cholinergic glial interactions between neurons and glial cells *via* α7nACh receptors could regulate neuroinflammation and oxidative stress, relevant to the treatment of several neurodegenerative diseases.

## Introduction

### Acetylcholine Receptors in the CNS

Acetylcholine (ACh) was one of the first identified neurotransmitters (Valenstein, 2002) and has been shown to modulate many physiological functions within the peripheral nervous system (PNS), the autonomic nervous system (ANS), and the central nervous system (CNS). In the CNS, ACh plays a crucial role in modulating diverse functions including cognition, attention, and arousal (English and Jones, 2012). This neurotransmitter is synthesized by choline acetyltransferase (ChAT) along with choline and acetyl coenzyme A as substrates. Signal transmission by this neurotransmitter is terminated through rapid hydrolysis of ACh into choline and acetic acid by acetylcholinesterase (AChE; English and Jones, 2012). ACh receptors are classified according to binding affinity to their ligands and can be divided into muscarinic (mAChR) or nicotinic receptors (nAChR).

*Muscarinic* *receptors*, named from M1 to M5, are metabotropic G protein-coupled receptors which are further subdivided according to the sub-type of associated G_α_ subunit. The M1, M3, and M5 receptors are coupled through G_q/11_ proteins to upregulate phospholipase C (PLC), inositol triphosphate, and intracellular calcium. M2 and M4 receptors deactivate adenylate cyclase and activate K^+^ channels through actions involving G_i_ (Akaike and Izumi, 2018). Muscarinic receptors are involved in cholinergic signal transduction in the CNS, autonomic ganglia, smooth muscles, and other parasympathetic end organs (Liu et al., 2007). They are also widely expressed in non-neuronal cells, such as epithelial, endothelial, muscle fibers, and CNS glial cells (microglia and astrocytes; Liu et al., 2007, 2016; Guizzetti et al., 2008).

*Nicotinic acetylcholine receptors*, which are the focus of this review, are ionotropic cation channels permeable to Na^+^ and K^+^ and are abundantly expressed in skeletal muscle and the CNS. These receptors belong to the cysteine-loop (Cys-loop) receptor ligand-gated ion channel superfamily and are extensively involved in cognitive and locomotive processes (Lester et al., 2004). Neuronal nAChRs are cation channels which can either be homopentamers formed of α7–α9 subunits, or heteropentamers formed of αβ subunits combinations including α2–α6 and β2–β4, or combinations of α9/α10 subunits (Dani and Bertrand, 2007). The most abundant and widely distributed nAChR subtypes in the human CNS are α4β2 and α3β4 heteromers, and α7 homomers (α7nAChR; Pym et al., 2005). α7nAChR is expressed in both neurons in the cortex and hippocampus, two structures related to cognition, attention, and memory tasks (Foucault-Fruchard and Antier, 2017) and non-neuronal cells, including microglia, astrocytes, oligodendrocytes, and brain endothelial cells (Dani and Bertrand, 2007; Cortes et al., 2017a,b; Morioka et al., 2018; Cao S. et al., 2019). Neuronal nAChRs have been studied extensively as potential drug targets for neurological disorders such as Alzheimer’s disease (AD) and Parkinson’s disease (PD; O’Neill et al., 2002; Lester et al., 2004). Recently, expression of these receptors in peripheral immune cells and CNS glial cells have attracted attention due to their involvement in neuroinflammation and neurodegenerative disease *via* the “cholinergic anti-inflammatory pathway,” as introduced later (De Jonge and Ulloa, 2007; Fujii et al., 2017; Takata et al., 2018).

### Structure and Function of the Cholinergic System

Cholinergic circuits in the CNS are involved in maintaining normal cognitive function and regulating signaling within the entire cerebral cortex. The four main mammalian brain regions with the highest expression of cholinergic neurons are: (1) the brainstem pedunculopontine and lateral dorsal tegmental nuclei projecting to the thalamus; (2) a subset of the thalamic nuclei; (3) the striatum interneurons, which are involved in suppressing dopamine release; and (4) the basal forebrain (BF) nuclei (Ballinger et al., 2016). The mammalian BF is a heterogeneous aggregate of subcortical structures including the medial septum, ventral pallidum, vertical and horizontal diagonal band nuclei, substantia innominata/extended amygdala, and peripallidal regions (Zaborszky et al., 2012; Agostinelli et al., 2019). Collectively, the BF serves as the major source of cholinergic projection neurons to the olfactory bulb, neocortex, hippocampus, and amygdala. The central cholinergic system helps regulate the vascularization of the brain (Sato and Sato, 1995; Van Beek and Claassen, 2011), and modulates cognition, specifically executive functions, attention, and memory (Levin and Simon, 1998; Ballinger et al., 2016; Prado et al., 2017; Solari and Hangya, 2018). The cholinergic neurons in the medial septum and the ventral diagonal band of the BF project to the hippocampus and are implicated in attention, short-term memory, and spatial learning (Boskovic et al., 2019). The cholinergic neurons of the brain stem (BS) project to the BF, the thalamus, and the ventral tegmental area (VTA), and are involved in regulating the waking state, behavioral responses as well as ocular saccades (Kobayashi and Isa, 2002; Newman et al., 2012; Mena-Segovia, 2016). Interestingly, cholinergic neurons appear to have a higher demand for energy production which may reflect a higher sensitivity to aging-related energy (glucose) deprivation (Szutowicz et al., 2013).

The cholinergic system modulates memory and hippocampal plasticity not only *via* neuronal cells but also *via* interactions with non-neuronal cells (e.g., microglia and astrocytes; Maurer and Williams, 2017). This suggests that microglia and astrocytes can respond directly to Ach *via* α7nAChRs and influence both short-term and long-term synaptic function and plasticity, contributing to cognition (Ben Achour and Pascual, 2010). BF cholinergic neurons release ACh in all regions of the hippocampus, which exhibit a high density of microglia and astrocytes, as well as nAChRs expressed in many hippocampal cell types (Maurer and Williams, 2017). It has been hypothesized that ACh acts as the decider between encoding and retrieval in memory-processing and thus, is implicated in suppressing old associations and inhibiting proactive interference (Easton et al., 2012; Maurer and Williams, 2017). Thus, rats with BF cholinergic lesions can perform equally to controls in a water maze if the location of the platform is not changed daily (Baxter et al., 2013). The rats with the intact cholinergic system can form new associations inhibiting previous associations, while rats with a lesioned BF tend to express a previously encoded association (the previous location of the platform), due to the lack of ACh in the hippocampus. The underlying neural events can be explained as follows: inputs to the CA1 region of the hippocampus come from two brain regions: entorhinal cortex layer 3 (associated with sensory perception/ “extrinsic input”) and the CA3 region of the hippocampus (associated with previously formed associations/ “intrinsic input”; Hasselmo, 2006; Easton et al., 2012). ACh reduces the recurrent pathway in the CA3 region *via* mAChRs activation on interneurons, and allow sensory inputs to be encoded, thus prioritizing encoding in novel contexts and allows working memory to be more efficient (Haam and Yakel, 2017; Maurer and Williams, 2017). Interestingly, a slow inhibition of dentate granule cells by septo-hippocampal release of ACh is not by a direct action on neurons but occurs rather by actions on both microglia and astrocytes *via* α7nAChRs. A proposed mechanism for neuronal-glial interactions *via* ACh and its impact on the hippocampus is that BF cholinergic projections release ACh and decrease cytokine release from microglia, as well as activate hilar astrocytes by activating the α7nAChRs (Hasselmo, 2006; Pabst et al., 2016). These activated astrocytes release glutamate, activating inhibitory interneurons, which in turn decrease firing from granule cells, leading to the decreased firing of CA3 pyramidal cells (Bezzi et al., 2004; Volterra and Meldolesi, 2005). Overall, this prevents interference of past associations on encoding, facilitating the formation of new memories (Pabst et al., 2016; Maurer and Williams, 2017).

In summary, it is established that the BF provides multiple inputs to the hippocampus and that these modulatory cholinergic inputs play an important role in cognitive functions. A substantial body of evidence suggests that the direct activation of neuronal nAChRs, and more specifically the α7 subtype, reverses cognitive deficits that arise due to disruption of the BF-hippocampal pathway (O’Neill et al., 2002). However, it is not only neurons that respond to ACh *via* nAChRs. Indeed, both hippocampal microglia and astrocytes express nAChRs, suggesting that ACh also affects the hippocampus through glial cell activation (Foucault-Fruchard and Antier, 2017). Hence, this review will primarily focus on the cholinergic anti-inflammatory and anti-oxidant pathways to memory processes during development, normal aging, and disease states, specifically *via* activation of glial α7nAChRs.

## The Functional Role of Glial Cells in the Immune Response of the CNS and Their Interaction During Neuroinflammation

As highlighted in a review from Herculano-Houzel (2014), after the discovery of glia cells, it was commonly thought that they massively outnumbered neurons and that their number was directly correlated with brain size. But in fact, it seems that the glia/neuron ratio increases as neuronal density decreases (Herculano-Houzel, 2014). A decrease in neuronal density is related to an increase in neuronal cell mass which is highly variable among species and brain structures in mammals, while the average glial cell mass is relatively conserved (Mota and Herculano-Houzel, 2014). Thus, the glia/neuron ratio varies among species and brain structures. For example, overall in the brain, this ratio is about 1 in humans and 0.54 in mice, and when looking at different brain structures in humans, it is about 0.23 in the cerebellum but is 1.48 in the gray matter of the cerebral cortex (Herculano-Houzel, 2014). The composition of the glial cells can also vary depending on the brain region considered, the sex and the age of the subject (Pelvig et al., 2008; Herculano-Houzel, 2014). This is important to keep in mind when considering the limitations from research conducted *in vivo* (with animal models) or *in vitro* (with cell culture) when translating these findings to humans.

This review will focus on two of the major glial cell types in the mammalian CNS: astrocytes and microglia, and their crosstalk, which is extremely important for neuronal development, regulation of synaptic transmission as well as protection of the CNS and maintenance of its homeostasis (Vainchtein and Molofsky, 2020). Glial cell activation physiologically occurs during CNS insults, such as inflammation, infection, or lesion, to facilitate neuroprotection. However, this activation can be dysregulated and promote chronic neuroinflammation and/or neurodegeneration.

*Microglia* are the innate immune resident cells of the CNS. In both developing and adult brains, these cells are critical for the maintenance of CNS homeostasis and perform several physiological functions, including regulation of neurogenesis, synaptic pruning, and production of trophic factors in both developing and adult brains (Ransohoff and El Khoury, 2016). In the presence of external stimuli that could negatively affect CNS homeostasis, microglia can acquire an activated phenotype to mediate a glial immune response that contributes to the development of pathological processes (Frank-Cannon et al., 2009; Kierdorf and Prinz, 2013). Possible causes for microglia activation include trauma, brain injury, stroke, apoptotic debris from dying neurons, aggregates of neurotoxic proteins, and pro-inflammatory cytokines. Microglia can transit from a resting or ramified state to an activated or primed amoeboid state, this transition is tightly regulated by molecular factors (e.g., CD200, CX3CR1 and TREM2) and minor changes in this signaling can potentially cause dysregulation of microglia homeostasis and pathology (Butovsky et al., 2014; Wolf et al., 2017; Deczkowska et al., 2018).

While the classical macrophage activation nomenclature (three functional states of polarized microglia phenotypes are postulated, M0, M1, and M2) has been adopted to define functional states of microglia, transcriptome analysis of microglia from animal models of neurodegeneration and aging suggests this classification is over-simplified (Chiu et al., 2013; Khakh and Sofroniew, 2015; Grabert et al., 2016; Ransohoff, 2016; Wes et al., 2016). Recent research suggests that microglia display phenotypes specific to changes in their microenvironment and surrounding CNS structures rather than showing only a distinct M1/M2 phenotype (Chiu et al., 2013; Khakh and Sofroniew, 2015; Grabert et al., 2016; Ransohoff, 2016; Wes et al., 2016). Historically, gene profiling data revealed that the molecular signature of the M0 phenotype was related to nervous system development and resembled adult microglia crucial in maintaining homeostasis (Butovsky et al., 2014; Sarlus and Heneka, 2017). The M1 phenotype appeared to express relatively high levels of inflammatory-related transcripts (*Ccl2*, *Ccl3*, and *Ccl5*), and upregulate cytokines like tumor necrosis factor (TNF), interleukin-1β (IL-1β), as well as nitric oxide (NO) and reactive oxygen species (ROS). The M2 phenotype expressed genes (e.g., *Insulin-like growth factor 1*) that promoted tissue development, neural cell renewal, and upregulated anti-inflammatory cytokines like IL-4 and IL10.

Microglia-mediated inflammation is essential to the primary acute CNS immune response; however, this acute response must be resolved to prevent chronic activation. To this end, microglia can release anti-inflammatory cytokines and tissue-repairing factors (Colton, 2009), and to reduce oxidative and nitrogen-induced stress by downregulating free radicals that accumulate in the interstitial space after trauma or in neurodegenerative diseases (Wagner et al., 2003; Moestrup and Møller, 2004). Dysregulation of this mechanism can be a breeding ground for the development of pathologies. A recent review summarizes how microglia priming renders aged microglia more responsive to proinflammatory stimuli, thus enhancing and prolonging their response to homeostatic disturbance and increased risk of neuronal loss and progression toward neurodegenerative disease (Wolf et al., 2017). Moreover, mouse models for aging and neurodegeneration account for senescent proinflammatory microglia that display morphological features and signaling pathways distinct from those microglia activated by the acute inflammation induced by lipopolysaccharides (LPS) in young mice (Holtman et al., 2015; Ransohoff and El Khoury, 2016). We have summarized the principal intracellular pathways involved in the regulation of microglial activity by highlighting their protective and detrimental roles in Table 1.

**Table 1:** Summary of protective and detrimental pathways of microglial signaling.

	Mediated by	Function	Effect			References
			Microglia activation	Inflammation	Neurodegeneration	
Protective microglia pathways	TGF-β *via* SMAD signaling	Neuroprotection, promote a quiescent state in microglia	↓	↓	↓	Abutbul et al. (2012)
	BDNF through JAK-STAT3 pathway	Neurotrophic	=	↓	↓	Zamanian et al. (2012) and Parkhurst et al. (2013)
	GDNF	Inhibition of microglia	↓	↓	↓	Rocha et al. (2012)
	NGF and NT-3 *via* p75	Downregulation of microglial activity	↓	=	=	Sobrado-Calvo et al. (2007) and Cragnolini et al. (2009)
	CD200-CD200R	Maintain microglia in a quiescent state	↓	↓	↓	Hernangómez et al. (2012); Shrivastava et al. (2012) and Oria et al. (2018)
	ORM2-CCR5 block CXCL4-CCR5	Inhibition of microglia	↓	↓	↓	Jo et al. (2017)
	TNF-TNFR2	Anti-inflammatory and neuroprotective	↑ anti-inflammatory cytokines	↓	=	Veroni et al. (2010) and Gao et al. (2017)
Detrimental microglia pathways	TNF-TNFR1	Microglia activation and neurotoxicity	↑	↑	↑ TNFR1-mediated neuronal loss	Fontaine et al. (2002)
	MD-1 (Ly86) protein	Innate immunity mediator	↑ increased proliferation	↑ chemokines CXCL10 and CCL2 production	↑	Jordão et al. (2019)
	IL-33 astrocytic expression	Pro-inflammatory	↑ chemokines expression	↑ recruitment of infiltrating macrophages in CNS	↑	Fairlie-Clarke et al. (2018)
	TLRs signaling	Pro-inflammatory	↑ cytokines production	↑	=	Kaminska et al. (2016)
	TREM2 myeloid cells receptor	Phagocytosis	↑ phagocytic activity	↑	↑	Wolfe et al. (2019)
	LCN2	Pro-inflammatory	↑ cytokines production and NO	↑	↑	Zamanian et al. (2012) and Bi et al. (2013)
	CCL2, CCL 21, CXCL10 *via* NF-kB signaling	Pro-inflammatory	↑	↑ recruitment of infiltrating immune cells in CNS	↑	Brambilla et al. (2005), Brambilla et al. (2009), Kim et al. (2014), and Mills Ko et al. (2014)

*Principal intracellular pathways involved in the regulation of microglial activity, according to the protective and detrimental roles of microglia. This table highlights the main function of microglia, followed by the specific effect on cellular activation and the effect on the inflammatory and neurodegenerative condition. ↑ and ↓ represent upregulation and downregulation, respectively, = means that the pathway does not affect that condition*.

*Astrocytes* are responsible for the maintenance and support of neurons. They are crucial in regulating oxidative stress thanks to the production of glutathione (GSH) and, according to the astrocyte-neuron lactate shuttle hypothesis, by glucose transformation into lactate which ensures energetic support of neurons (Sidoryk-Wegrzynowicz et al., 2011). Astrocytes can also regulate innate and adaptive immune responses in the CNS by controlling the activation and immune cell trafficking and are immune-competent cells able to detect danger signals and respond *via* secretion of cytokines and chemokines and activate adaptive immune defenses (Farina et al., 2007). In reaction to harmful stimuli to the CNS, astrocytes can respond with proliferation, migration, hypertrophy, and increased production of glial fibrillary acidic protein (GFAP). Indeed, inflammatory stimuli from the microenvironment can shift the role of astrocytes from a protective “neuronal-focused” state to an inflammatory-focused state, also called “neuronal-neglecting.” Activated astrocytes were originally divided into two groups—A1 and A2. A1 astrocytes promote inflammation, lose the ability to protect neurons and control synaptogenesis, become phagocytic, and lead to neuronal loss through the NF-kB pathway. A2 astrocytes upregulate neurotrophic factors promoting neuronal survival *via* Janus Kinase/Signal Transducer and Activator of Transcription 3 (JAK-STAT3) signaling, which leads to the production of neurotrophic factors like brain-derived neurotrophic factor (BDNF; Li K. et al., 2019). However, as with microglia, the A1/A2 astrocytic phenotypes do not exactly account for the *in vivo* complexity of astrocyte activation. Rather, there appears a continuous spectrum of astrocytic activation (or astrogliosis) with a gradient in morphological as well as functional changes based on the nature and severity of the stimuli (Sofroniew, 2015). Also, heterogeneity in astrocyte morphology and physiology (Matyash and Kettenmann, 2010) may affect glial responses to specific inflammatory stimuli (e.g., an early or late activation of astrocytes can modulate a disease onset and progression). A summary of both pro- and anti-inflammatory astroglia intercellular pathways is provided in Table 2.

**Table 2:** Summary of protective and detrimental pathways of astroglial signaling.

	Mediated by	Function	Effect			References
			Microglia activation	Inflammation	Neurodegeneration	
Protective microglia pathways	Glycoprotein gp130 Signal transducer for IL-6 cytokine family	Astrocyte’s survival	↑ astrocytes-mediated apoptosis	=	↓	Haroon et al. (2011)
	TGFβ signaling	Immuno-suppressive	↓ proliferation	↓ inhibition of NF-kB signaling	↓	Cekanaviciute et al. (2014)
	IFNyR through T and NK cells	Inflammatory regulation and immuno-suppressive	↓ proliferation	↓ down-regulation of iNOS, TNF, IL-10, and IL-27	↓	Hindinger et al. (2012)
	NrF2 pathway	Anti-inflammatory and antioxidant	↑ expression of antioxidant genes	↓ suppression of cytokines	↓	Patel et al. (2017)
	BDNF through JAK-STAT3 pathway	Anti-inflammatory and neurotrophic	↓	↓ suppression of cytokines	↓	O’Callaghan et al. (2014)
Detrimental astrocyte pathways	BDNF through TRkB receptor and NF-kB pathway	Pro-inflammatory	↑ increase of NO released by astrocytes	↑	↑	Colombo et al. (2012)
	NFkB recruitment after IL-17 binding to its receptor	Release of pro-inflammatory cytokines and chemokines	↑	↑ *via* astrocytic IL-17 pathway	↑	Brambilla et al. (2005)
	CCL2, CCL 21, CXCL10 *via* NF-kB signaling	Pro-inflammatory	↑	↑ recruitment of infiltrating immune cells in CNS	↑	Brambilla et al. (2005, 2009), Kim et al. (2014), and Mills Ko et al. (2014)
	CXCL12	Pro-inflammatory	↑	↑ pro-inflammatory cytokines production	=	Bezzi et al. (2001)
	VEGF	Control of vascular permeability (BBB integrity)	=	↑ recruitment of infiltrating immune cells in CNS	↑	Argaw et al. (2012)

*The table reports the principal intracellular pathways involved in the regulation of astrocytes activity by dividing the protective and the detrimental role. It highlights the main function of astroglia, followed by the specific effect on cellular activation and the effect on the inflammatory and neurodegenerative condition. ↑ and ↓ represent upregulation and downregulation respectively, = means that the pathway does not affect that condition*.

The maintenance of CNS integrity depends on glial cells and their well-orchestrated interactions. As the balance between protective vs. detrimental function of glial cells is regulated by surface and cytoplasmic proteins, transcription factors, and released mediators, it is crucial to highlight the crosstalk between distinct neuroinflammatory pathways and identify their shared effector mechanisms (Colombo and Farina, 2016). There is evidence supporting a faster response by microglia to stimuli, showing a higher phagocytic activity after IFN-γ stimulation compared to astrocytes (Magnus et al., 2002). During CNS injury there is a rapid release of cytokines by microglia, while the astrocytic response seems to be delayed (Zhang et al., 2009, 2010). These findings are supported by the high dynamicity of microglia typical of their resting state; indeed, microglia appear to be the dynamic surveillants of CNS (Nimmerjahn et al., 2005; Ransohoff and Cardona, 2010; Gomez-Nicola and Perry, 2015) and their activation can trigger and modulate the different responses of surrounding glial cells. Microglial dynamics can be stimulated by factors like ATP and other nucleotides or reduced by factors including fractalkine, also known as chemokine CX3CL1 (Liang et al., 2009). Recent evidence supporting the role of microglia in regulating the astrocytic response (Jha et al., 2019) indicates a reactive astrocytic phenotype through the release of IL-1α, TNF-α, and complement component 1 (C1q; Liddelow et al., 2017). However, microglia-astrocyte interactions *via* purinergic signaling are crucial to switch astrocytes toward a neuroprotective phenotype (Shinozaki et al., 2017). In return, astrocytes can also regulate microglial phenotypes and functions through numerous astrocyte-derived factors, such as cytokines, chemokines, complement proteins, Ca^2+^, and other inflammatory mediators (Tanuma et al., 2006; Jha et al., 2019). Moreover, microglial activation can also be modulated by astrocyte- released glial cell line-derived neurotrophic factor (GDNF), cerebral dopamine neurotrophic factor (CDNF), and BDNF. Astrocyte-derived GDNF in particular has been shown to control midbrain microglial activation and prevent neurodegeneration through inhibition of neuroinflammation (Rocha et al., 2012). Of particular interest is the role of two proteins belonging to the lipocalins family which astrocytes, observed in mouse and rat models, can mediate microglial activation: orosomucoid 2 (ORM2) binds CC chemokine receptor 5 (CCR5) on microglia and blocks the interaction between chemokine (C-X-C motif) ligand 4 (CXCL4)-CCR5, which is crucial for their activation (Jo et al., 2017). Lipocalin-2 opposes ORM2 function and enhances inflammatory activity (Lee et al., 2009) by the production of pro-inflammatory mediators including IL-1β, IL-6, TNF-α, and NO (Zamanian et al., 2012; Bi et al., 2013).

Considering that most cells in the brain, including astrocytes (Liddelow et al., 2017) and microglia (Füger et al., 2017), have a long lifespan, it is plausible that the accumulation and overstimulation of inflammation can trigger multiple cumulative molecular modifications (e.g., telomere shortening, DNA damage, epigenetic modifications, lysosomal dysregulation) that eventually contribute to cellular senescence and loss of function (Desplats et al., 2019).

## The Interaction Between Cholinergic Neurons and Glial Cells—the Cholinergic Anti-inflammatory Pathways

### Anatomical Evidence for the Connection Between Cholinergic Neurons and Glial Cells

In the CNS, both microglia and astroglia express muscarinic and nicotinic AChRs, which can be composed of different sub-units depending upon the brain area and the species considered (André et al., 1994; Guizzetti et al., 1996; Xiu et al., 2005; Rock et al., 2008; Morioka et al., 2014; Pannell et al., 2016). This might raise the question of the validity of rodent models when studying the role of nAChR and mAChR on neuroinflammation. To our knowledge, more studies are still needed to fully characterize the expression profile of mAChR and nAChR subunits in both microglia and astrocytes. The currently known subunits expressed in humans, rats, and mice have been summarized in Table 3. Microglia express a variety of neurotransmitter receptors that help to intercede bidirectional communication with neurons, including glutamate, GABA, norepinephrine, cannabinoid, and ACh receptors (Liu et al., 2016). In humans, microglia express the α3, α5, α7, and β4 subunits of nAChR, while rat cortical microglia exclusively express α7nAChRs (Morioka et al., 2018). A limited number of studies using electron microscopy indicate a close relationship between glia and cholinergic neurons. In the septal complex of the BF, the dendrites, axon, and soma of cholinergic neurons are mostly surrounded by astrocytic processes (Milner, 1991). In the laterodorsal and pedunculopontine tegmental nuclei of the brainstem, cholinergic neurons receive a large amount of synaptic input, with approximately one-quarter of their somatic surface covered by astrocytic processes (Honda and Semba, 1995). Expression levels of α7nAChR have been investigated in various inflammatory models. On one hand, an increase of α7nAChR expression in both microglia and astrocytes was reported in cerebral ischemia in rats, leading to microglial activation and pro-inflammatory cytokine production (Niranjan et al., 2012; Wu L. et al., 2014; Colás et al., 2018). This was counteracted by nicotine treatment, which reduced pro-inflammatory cytokine production as well as microglial activation, and also prevented neuronal death in the CA1 region of the hippocampus in rats (Guan et al., 2015). Also, activation of α7nAChR by a pre-treatment with a positive allosteric modulator (PAM) reduced LPS-induced expression of the pro-inflammatory markers IL-1β, TNF-α, and the microglial activation marker cluster of differentiation 11b (CD11b) in the hippocampus and prefrontal cortex (PFC) of mice, and even blocked LPS-induced anxiety-like behaviors (Abbas et al., 2017; Alzarea and Rahman, 2019). On the other hand, neuroinflammation induced by the intra-cerebroventricular injection of an inflammatory soup containing prostaglandin E2 (0.2 mM), serotonin (2 mM), bradykinin (2 mM), and histamine (2 mM), in a rat model can also be linked with a decrease in the expression level of α7nAChR in the hippocampus, which can be reversed by the treatment with an α7nAChR agonist (Liu et al., 2018).

**Table 3:** Summary of known AChR subunits to be expressed in glial cells in human, rat, and mouse.

		Microglia		Astrocytes	
		Subunits	Origin	Subunits	Origin
Human	*nAChR*	α3, α5, α7, β4	Fetal brain (Rock et al., 2008)	α7	Hippocampus and entorhinal cortex (Teaktong et al., 2003)
	*mAChR*	M5	Levey (1996)	M2, M3, M5	Fetal brain (Guizzetti et al., 1996)
Rat	*nAChR*	α7, α4, β2	Neonatal Cortex (Morioka et al., 2014) Adult brain (Martín et al., 2015)	α4, α7, β2, β3	Neonatal brain (Xiu et al., 2005)
	*mAChR*	M1	Adult cortex and hippocampus (Huang et al., 2016)	M2, M3, M5	Cell line 132 1N1 (Guizzetti et al., 1996)
Mouse	*nAChR*	α7	Cerebral cortices (Shytle et al., 2004)	α7 β4	Neonatal brain (Patel et al., 2017) The hippocampus of the adult brain (Gahring et al., 2004)
	*mAChR*	M1, M2, M3, M4, M5	Neonatal and adult whole brain (Pannell et al., 2016)	M1, M3	Neonatal cerebral hemisphere, mesencephalon, and medulla-pons (André et al., 1994)

*This table presents the different subunits of nAChR and mAChR that have been found until today (and to the best of our knowledge) in microglia and astrocytes in humans, rat, and mice*.

### Functional Evidence for a Connection Between Cholinergic Neurons and Glial Cells

Various electrophysiological studies have demonstrated how glial responses to cholinergic activation are the result of a balance between the hyperpolarizing action of ACh and the opponent modulation of glutamate and GABA from surrounding neurons. A study of rat hippocampal slices showed that cholinergic stimulation of glial cells increases intracellular Ca^2+^ mobilization (Araque et al., 2002). Understanding Ca^2+^ transmission in neuron and astrocyte interactions and effects on cholinergic activation is essential for pathologies characterized by dysfunctional cholinergic signaling (Parpura et al., 1994; Verkhratsky and Kettenmann, 1996; Bezzi et al., 1998; Perea and Araque, 2005; Verkhratsky, 2006). *In vivo* studies of cats have found that during activation states associated with ACh transmission, cortical glial cells are hyperpolarized through the interaction between membrane ACh and muscarinic AChRs (Seigneur et al., 2006). This interaction, observed in mice, causes neuronal depolarization that leads to the release of glutamate and GABA from neurons (Czarnecki et al., 2014; Takács et al., 2018), which can in turn cause depolarization of glial cells. Besides, glial cells can also be influenced by the release of K^+^ provoked by the excitation of neighboring neurons, and this could be related to increased membrane resistance and decreased membrane capacitance in glial cells (Seigneur et al., 2006). *In vivo* cat and *in vitro* mouse models have observed decreased glial capacitance during cholinergic activation reflects several phenomena, such as osmotic changes and modifications of the gap junction permeability (Amzica and Neckelmann, 1999; Hernández-Balaguera et al., 2018), which drive glial shrinkage and the consequent expansion of extracellular space resulting in a decrease of the overall K^+^ concentration. These studies illustrate a functional electrophysiological relationship between cholinergic neurons and astrocytes, highlighting the importance of this axis.

Microglial activation following LPS administration in the hippocampus decreases BDNF release by astrocytes in the CA1 region of rats (Tanaka et al., 2006). Chronic nicotine exposure activates nAChRs in the rat hippocampus and upregulates BDNF in this brain region (Kenny et al., 2000). Interestingly, cholinergic neuronal loss in the BF leads to a subsequent reduction of BDNF in the hippocampus of mice (Turnbull and Coulson, 2017). Furthermore, BDNF increases α7nAChR density on hippocampal neurons, and activation of α7nAChRs upregulates BDNF in the rat hippocampus (Freedman et al., 1993; Massey et al., 2006). However, there is no direct evidence linking microglial α7nAChR activation and BDNF levels. It is also interesting that pharmacological activation of α7nAChRs in the hippocampus (CA1 and CA3 regions) increases hippocampal long-term potentiation (LTP) through long-lasting increases in calcium activity in wild-type, but not in α7nAChR knockout mice (Gu et al., 2012). The α7nAChR is reported to be highly permeable to Ca^2+^ (Shen and Yakel, 2009), and microglial α7nAChR stimulation also increases cytosolic Ca^2+^ (Takata et al., 2018). Together, this suggests activation of α7nAChR on neurons as well as microglia can facilitate LTP.

After brain insult, ATP is released from injured cells and activates microglia which, thanks to the interaction with the purinergic P2X7 receptor, starts to release molecules to protect neurons of the neonatal rat brain *in vitro* (Suzuki et al., 2004). In particular, P2X7 receptor stimulation has a role in neuronal protection against glutamate toxicity mediated by small amounts of TNF-α released by microglia, while LPS treatment mediates TNF-α production that leads to neuroinflammation (Suzuki et al., 2006). Considering that ACh can elicit glutamate release through presynaptic α7nAChRs, and ATP is a co-transmitter of ACh, Patti et al. (2006) investigated the role of the P2X7 receptor in this interaction in the rat neocortex. They found P2X7 receptors and α7nAChRs co-existed and interacted on glutamatergic terminals where P2X7 exerts a permissive role on the activation of α7nAChRs, suggesting regulation of ATP-mediated signaling. A later work observed colocalization of the P2X7 receptor with astrocytes and microglia, but not neurons, and proposed a role for these receptors in glial apoptotic and proliferative functions after ATP stimulation (Oliveira et al., 2011). Furthermore, it is generally recognized that healthy neurons and astrocytes can regulate microglia-mediated innate immune responses *via* activation of *α*7nAChR and purinergic P2X7 receptors (Suzuki et al., 2006). Indeed, the hyperactivation of the P2X7 receptor by ATP drives microglia toward a reactive phenotype and can increase pro- or anti-inflammatory microglia markers (Parisi et al., 2016). An example, observed in embryonic rat pups *in vitro*, is the increased formation of membrane vesicles in microglia containing IL-1β after ATP stimulation mediated by the P2X7 receptor, which leads microglia to promote inflammation (Bianco et al., 2005).

The anatomical and electrophysiological evidence for a connection between the cholinergic system and glial cells supports further investigation into the role of aging and chronic microglial activation on their bidirectional relationship. Possibly, the loss of cholinergic connections, resulting from either damaged neurons or astrocytes may lead to hyperactive microglia, which then causes persistent neuroinflammation and augmentation of neurodegeneration.

### The Anti-inflammatory Role of the Cholinergic System

The existence of a *“cholinergic anti-inflammatory pathway”* (CAP) in the CNS, mediated by the activation of α7nAChR on microglia, was first proposed by Shytle et al. (2004). They demonstrated that pre-treatment of cultured murine-derived microglial cells with ACh and nicotine inhibited LPS-induced TNF-α release, which was attenuated by α7 selective nicotinic antagonist, α-bungarotoxin (Shytle et al., 2004). Thus, the α7nAChR subunit was confirmed as essential in the endogenous cholinergic anti-inflammatory pathway for inhibiting cytokine synthesis by macrophages. This was later confirmed by several other *in vivo* and *in vitro* studies demonstrating the existence of the cholinergic anti-inflammatory pathway in the CNS, which appears to mainly rely on the activity of nicotinic receptors and more specifically, α7nAChR (De Jonge and Ulloa, 2007; Kalkman and Feuerbach, 2016; Hoover, 2017).

Neuroinflammation *in utero*, due to maternal infection during pregnancy, may contribute to fetal brain injury and life-long risk of neurodevelopmental defects (Al-Haddad et al., 2019). Glial cells, both microglia and astrocytes, play a pivotal role in the risk of neurodevelopmental defects but the exact mechanisms are poorly understood. However, recent studies have shed new light on the inflammatory phenotypes of fetal glia involved in life-long neurological disabilities (“second hit” hypothesis). Using an *in vivo–in vitro* model of developmental programming of neuroinflammation induced by LPS, Cao et al. (2015) demonstrated that the inflammatory microglial phenotype acquired by exposure to LPS in sheep fetus (*in vivo*) sustained and potentiated a pro-inflammatory phenotype *in vitro* upon re-exposure to LPS. They also confirmed the downregulation of heme oxygenase (HO)1 gene, a key gene of iron homeostasis, as well as an upregulation of gluconeogenesis (energy conserving) fructose-1,6-biphosphate (FBP) gene, in second hit microglia compared to single hit microglia (Cao et al., 2015). This suggests an interplay of inflammatory and metabolic pathways and immunological and metabolic memory of the prior-inflammatory insult relevant to neuronal development. A study expanding the work of Cao et al. (2015) used primary fetal sheep microglia cultures re-exposed to LPS in the presence of a selective α7nAChR agonist (AR-R17779) or antagonist (α-bungarotoxin; Cao et al., 2015; Cortes et al., 2017b). The microglial α7nAChR agonist reversed the pro-inflammatory microglial phenotype acquired *in vitro* by LPS stimulation while blocking α7nAChR potentiated the pro-inflammatory microglial phenotype (Cortes et al., 2017b). In this study, a link between iron homeostasis and microglia α7nAChR has also been proposed (Cortes et al., 2017b); iron accumulation and toxicity are related to oxidative stress, which is found in numerous neurodegenerative diseases (Gaasch et al., 2007).

A very recent study extended this investigation to examine the role of α7nAChR in fetal sheep astrocytes (Cao M. et al., 2019) and showed that the pro-inflammatory transcriptome astrocyte phenotype acquired *in vivo* or *in vitro* by LPS stimulation was reversed by α7nAChR agonist. Conversely, α7nAChR inhibition potentiated the pro-inflammatory astrocytic phenotype and pro-inflammatory signaling pathways NF-κB and STAT3 (Cao M. et al., 2019). The exact mechanism by which activation of nAChR on astroglia or microglia can lead to an anti-inflammatory effect is still under investigation. A recent study applied ACh to rat hippocampal neuron-microglia co-cultures to confirm its anti-inflammatory properties in response to microglia-derived neuroinflammation (Li L. et al., 2019). A higher concentration of ACh markedly inhibited the LPS-induced microglial inflammatory response by decreasing pro-inflammatory factors and inhibiting hippocampal neuronal apoptosis, showing that ACh has concentration-dependent anti-inflammatory and neuroprotection properties in this model (Li L. et al., 2019). They also demonstrated, by genetic knockdown of α7nAChR, that both the anti-inflammatory and neuroprotective abilities of ACh depend on microglial α7nAChR signaling (Li L. et al., 2019). In a model of oxaliplatin-induced neurotoxicity in co-cultured neurons and astrocytes, the application of an α7nAChR agonist increased neuron viability by preventing caspase-3 activation, increased glutamine synthetase expression levels, and increased the production of the anti-inflammatory cytokine TGF-β (Di Cesare Mannelli et al., 2015). Thus, the activation of cholinergic receptors could not only have an anti-inflammatory effect but also an antioxidant and neuroprotective role, as these three phenomena are highly interconnected. Indeed, ACh has been suggested to exert both anti-inflammatory and neuroprotective properties in several neurodegenerative disorders (Gallowitsch-Puerta and Pavlov, 2007; Martelli et al., 2014).

In astrocytes, activation of α7nAChRs has an anti-inflammatory effect. This is mediated by the activation of nuclear factor erythroid 2-related factor 2 (Nrf2), which allows the expression of several antioxidant genes (HO1, thioredoxin reductase 1, glutamate-cysteine ligase catalytic subunit) and the inhibition of the NF-κB pathway with a decrease of the expression of p50, of an inhibitor of κBα (IκBα) phosphorylation and NFκB nuclear translocation (Patel et al., 2017).

The anti-inflammatory action of α7nAChR in microglia is also mediated *via* the Nrf2-HO1 pathway (Parada et al., 2013). Moreover, the activation of α7nAChR in microglia decreases the phosphorylation, and therefore the activation, of the p38, p44/42 and c-jun N-terminal kinase (JNK) MAP kinases (Shytle et al., 2004; Suzuki et al., 2006), which are involved in neuroinflammation (Koistinaho and Koistinaho, 2002; You et al., 2018; Zhao et al., 2018). Interestingly, it also increases the expression of cyclooxygenase-2 (COX-2) and prostaglandin E_2_ (De Simone et al., 2005), but the latter has a complex role in neuroinflammation and can either be pro- or anti-inflammatory (Levi et al., 1998; Zhang and Rivest, 2001).

Some evidence suggests that α7nAChR can function as a metabotropic receptor by activating the PLC-inositol-3-phosphate (IP3) pathway, which triggers the release of Ca^2+^ from intracellular stores, and can reduce the release of TNF-α triggered by LPS -induced neuroinflammation in microglia (Suzuki et al., 2006; Brawek and Garaschuk, 2013; Hua et al., 2014). Experiments in neuroblastoma support the hypothesis that α7nAChR can also be a metabotropic receptor. α7nAChR is physically connected with JAK2, phosphatidylinositol-3-kinase (PI3K), and Fyn, and its activation leads to neuroprotection against amyloid-β (Aβ)-induced toxicity *via* the PI3K, leading to the upregulation of neuroprotective factors such as B-cell lymphoma 2 (Bcl-2; Kihara et al., 2001; Shaw et al., 2002). This neuroprotective effect is coherent with the decrease in LPS-induced caspase activation that has been shown in astrocytes after activation of α7nAChR (Patel et al., 2017).

An important structure to discuss when considering the CAP is the vagus nerve. Peripherally, this nerve is involved in a neuroimmune reflex which can attenuate TNFα production during endotoxemia, giving the vagus nerve promising clinical implications during sepsis (Bonaz et al., 2016). While the efferent arm of this reflex arc has been well described, the implication of a vagal-mediated central CAP has yet to be fully elucidated. Visceral afferents of the vagus nerve in the gut may be stimulated by cytokines, namely IL-1, to induce a neuroimmune reflex. This information is received by the nucleus of the solitary tract where a synaptic relay occurs with the dorsal motor nucleus of the vagus. Efferent vagal fibers will then communicate with splenic lymphocytes and macrophages to reduce the production of pro-inflammatory cytokines *via* the α7nACh receptor (Bonaz et al., 2016). Recent evidence suggests that this neuroimmune signaling may go on to higher centers, including the BF, to mediate a centralized CAP. Both afferent and efferent fibers of the vagus nerve have been shown to communicate with limbic structures, where afferents increase cholinergic signaling in the BF (Broncel et al., 2018; Suarez et al., 2018). This BF cholinergic signaling reduces pro-inflammatory cytokines both centrally and peripherally in an effect which requires an intact vagus nerve (Zhai et al., 2017). Vagal afferents may also influence cholinergic signaling through norepinephrine production from the locus coeruleus (LC), which in turn promotes ACh production from the Nucleus Basalis of Meynert (Kaczmarczyk et al., 2018). In light of these findings, the BF has been proposed as a higher integrative center for neuroimmune signaling by way of the vagus nerve. Recent studies have provided evidence for the anti-inflammatory role of vagal cholinergic signaling in the CNS. By using an *in vivo* model of near-term ovine fetuses, Frasch et al. (2016) described how microglial activation, induced by acidosis, was less prominent in animals with higher vagal afferent signaling and that this effect is likely mediated by the α7nACh receptor. The higher vagal activity was associated with a higher count of α7nACh receptors in neural tissue, suggesting a relationship between vagal innervation and α7nACh receptor expression. Reduced microglial activation and attenuation of pro-inflammatory cytokines were observed with vagal nerve stimulation (VNS) during peripheral LPS challenge in mice (Meneses et al., 2016). Morphological signs of microglial activation in a murine model of AD were reversed by VNS (Kaczmarczyk et al., 2018). Further, VNS used in a murine model of stroke provided a neuroprotective effect that was mitigated by α7nACh receptor antagonists (Lu et al., 2017). While future research is required to better understand the neuroimmune signaling pathways between the BF and afferent vagus nerve fibers, it appears vagal activity may have significant anti-inflammatory properties in the CNS by way of cholinergic signaling.

In summary, both astrocytes and microglia are involved in the cholinergic anti-inflammatory pathway, *via* α7nAChR expression, resulting in anti-inflammatory, antioxidant, and neuroprotective effects. We hypothesize that the loss of cholinergic profiles resulting in decreased α7nAChR activation on glial cells has a significant impact during aging and contributes to the development of the neurodegenerative disease.

## Implications of Cholinergic-Glial Interactions in Aging and Neurodegenerative Disorders

### Physiological Aging

Aging is a physiological process accompanied by a decline in brain function, reduction in synaptic plasticity, and changes in neurotransmission and receptor availability in the CNS which may affect cognitive performance (Li et al., 2001; Mahncke et al., 2006). Hallmarks of brain aging include increased oxidative stress and inflammation (Lee et al., 1999; Godbout et al., 2005), due to the accumulation of free radicals and enhanced expression of pro-inflammatory cytokines, including IL-1β and IL-6 (Sierra et al., 2007; Kuzumaki et al., 2010), combined with decreased in anti-inflammatory cytokines such as IL-10 and IL-4 (Ye and Johnson, 2001; Nolan et al., 2005). Resident glial cells are key contributors to the age-related shift in the inflammatory profile of CNS. Histological examination of postmortem brain samples reveals microglial de-ramification and the shortening of cellular processes that are related to the activated state of microglia (Streit et al., 2004). Furthermore, microglia exhibit a “primed” phenotype, indicating an enhanced inflammatory response to an immune stimulus (Perry and Holmes, 2014). This can be accompanied by upregulation of genes associated with antigen presentation, like major histocompatibility complex (MHC) II and CD68 (Frank et al., 2006; VanGuilder et al., 2011), suppression of anti-inflammatory proteins, such as IL-10 and CD200 (Frank et al., 2006), and rise of pro-inflammatory cytokines as IL-1β (Henry et al., 2009). Microglia also decrease their mobility with age, becoming less reactive in surveying the CNS microenvironment, and reducing the resolution of an established inflammatory state (Damani et al., 2011). Astrocytes move from a resting/flat morphology towards a stellate state (VanGuilder et al., 2011), and acquire an activated profile indicated by increased expression of GFAP (Zamanian et al., 2012; Liddelow et al., 2017). Reactive astrocytes also exhibit upregulated MHCI (Mangold et al., 2017), and chemoattractants for infiltrating immune cells such as CXCL10 and CXCL5 (Boisvert et al., 2018; Sorensen et al., 2018). Interestingly, the receptor for CXCL10 is CXCR3 which is a microglial marker, suggesting communication between astrocytes and microglia that affects aging (Rothhammer et al., 2018).

Glial inflammatory profiles are regulated by ACh through α7nAChRs on astrocytes and microglia, to control the production of inflammatory cytokines (Niranjan et al., 2012; Kalashnyk et al., 2014). Thus, cholinergic transmission appears crucial in regulating glial cell inflammatory profiles during aging. During development and aging, there are changes to the profiles of nicotinic and muscarinic receptors. In a study using human brain tissue from 24 weeks of gestation to 100 years, Court et al. (1997) found the highest level of nAChR expression in the late fetal stage in the hippocampus, entorhinal cortex, and presubiculum. In the hippocampus, this is followed by a considerable decrease within the first 6 months of life and a slight decrease during aging (Court et al., 1997). Muscarinic ACh receptor expression slightly decreased with age in the hippocampus and the subicular complex (Court et al., 1997). This age-related decrease in cholinergic receptor expression may contribute to age-related cognitive impairment (Dumas and Newhouse, 2011). Indeed, a reduction in cholinergic transmission appears to affect the protective and supportive role of glial cells to neurons, and this is mediated by ACh. An example is the expression of GDNF stimulated by the activation of astrocytic α4β2 and α7nAChRs, whereby astrocyte-derived GDNF promotes the inhibition of microglia activation and neuronal protection (Rocha et al., 2012; Konishi et al., 2014). The neuroprotective effect of nAChR can be mediated by different subunits in different brain regions, for instance, α4β2-type nAChRs are involved in nicotine-mediated protection in the cortex and striatum (Ryan et al., 2001; Laudenbach et al., 2002) while α7 subunits are involved in hippocampal nAChRs (Messi et al., 1997; Dajas-Bailador et al., 2000). Studies on aged β_2_ subunit knockout mice show neocortical hypotrophy, loss of pyramidal neurons in the CA3 field, and Astro- and microgliosis in neocortex and CA1–3 hippocampal fields, suggesting that chronic loss of β_2_ subunit removes the protective role of glial cells against neurodegenerative processes during aging in the mouse brain. The same mice also showed impaired spatial learning (Zoli et al., 1999; Caldarone et al., 2000), implying that nAChRs contribute to both neuronal survival and maintenance of cognitive performance during aging (Picciotto and Zoli, 2002).

A SPECT imaging human study involving 47 healthy female and male volunteers from 18 to 85 years old, revealed an inverse correlation between age and regional β2 nAChR availability in several brain regions including the thalamus, frontal, parietal, and anterior cingulate cortices (Mitsis et al., 2007). These results confirmed what was observed in postmortem human samples using RT-PCR, where mRNA expression of α4 and β2 subunit nAChRs decreased with age in the frontal cortex, while in the hippocampus, only β2 significantly decreased with age (Tohgi et al., 1998). The age-related changes also affect the α7nAChR: binding experiments with [^125^I]α-bungarotoxin, the specific marker of this nAChR subtype, showed a higher expression of the α7 subunit in several brain areas in the human fetal sample (9–11 weeks of gestation) compared to middle-aged (28–51 years) and aged (68–94 years) samples (Falk et al., 2003). However, opposing evidence has been reported: a PET imaging study with 25 healthy volunteers aged from 21 to 86 years showed a positive correlation between α7nAChRs distribution and age in all the brain regions investigated, including the thalamus, striatum, hippocampus, cerebellar cortex, temporal cortex, occipital cortex, cingulate cortex, frontal cortex, and parietal cortex, suggesting an increase in cerebral α7nAChRs during healthy aging (Coughlin et al., 2018). Rodent data may shed light on these seemingly contradictory results, as not all nAChR subunits appear equally affected by age. Adult (12–14 months) and aged (24–28 month) mice from two strains (C57BL/6 and CBA/J) showed different trajectories of α4 and α7 expression with age (Gahring et al., 2005; Utkin, 2019). CBA/J mice demonstrated a considerable decrease in α4 and α7 expression with age, while C57BL/6 mice showed a slight decrease in α4 and a reduction in β4 nAChR with age, suggesting that the interpretation of age-related AChR changes should be done very carefully, preferentially using a large group of individuals (Gahring et al., 2005; Utkin, 2019). Moreover, electrophysiological studies using whole-cell patch-clamp recordings in mouse brain slices highlighted differences in the contribution of nAChR subunits to ACh-induced inward currents across aging (Christensen and Kohlmeier, 2016). In younger animals, this was predominantly mediated by the nAChR containing β2 and/or β4 subunits, however, in neurons from older animals, the currents conducting by nAChRs containing the β2 and/or β4 and the α7 subunits were similar (Christensen and Kohlmeier, 2016).

All these findings support the cholinergic hypothesis of age-related cognitive dysfunction and underline the importance of a better characterization of the contribution of nAChR subunits during aging (Dumas and Newhouse, 2011; Utkin, 2019). One of the possible future directions in the field of studying normal aging could be to find a specific correlation between the loss of different cholinergic receptor subunits and resulting cognitive impairment. Eventual findings could be very helpful in understanding not only physiological aging but also the pathogenesis of the age-related neurodegenerative disease. Physiological aging is also associated with active clearance of accumulated neurotoxic proteins, like amyloid and tau, from and around neurons by microglia (Clayton et al., 2017). The failure of this process leads to the formation of aggregates, typical of AD and other forms of dementia, and dysfunction of the neuron to glia communication. Indeed, there is the age-related loss of cholinergic function driven by dendritic, synaptic, and axonal degeneration, a decrease in neurotrophic support, and impairment in glial intracellular signaling (Clayton et al., 2017). The aging process alters the expression of muscarinic and nicotinic receptors for ACh, contributing to impaired crosstalk between neurons and surrounding glial cells, which may be responsible for dysfunction leading to neurodegenerative processes instead of normal brain aging (De Keyser et al., 2008; Sofroniew, 2009).

### Pathological Aging and Neurodegenerative Disorders

Inflammation and aging appear closely linked and microglial activation has been shown to increase with age (Bachiller et al., 2018). While low levels of inflammation correlate with healthy brain function (Walker et al., 2017) and longevity (Arai et al., 2015), high levels of inflammation during aging are likely to disrupt brain homeostasis and the physiological equilibrium between the pro- and the anti-inflammatory response of microglia and astrocytes.

Basic signaling between astrocytes and neurons is preserved in the early stages of aging; however, in a mouse model of AD, this communication mechanism is dysfunctional, affecting astrocyte Ca^2+^ signaling, which is crucial for normal brain activity (Gómez-Gonzalo et al., 2017). This confirms what was previously observed regarding astrocytic Ca^2+^ signaling during age-related neurodegeneration: mouse cortical astrocytes near Aβ plaques demonstrate enhanced Ca^2+^ excitability (Kuchibhotla et al., 2009). Moreover, Ca^2+^ levels are increased in hippocampal slices acutely treated with Aβ oligomers (Pirttimaki et al., 2013; Talantova et al., 2013). While both microglia and astrocytes respond to perturbations that lead to an activated state, this mechanism needs to be tightly regulated to avoid glial cell overstimulation, which could result in a failure to return to a resting basal state and leading to cellular dysfunction (Van Rossum and Hanisch, 2004; Biber et al., 2007). Physiological aging leads to activated microglia and increased glial neuroinflammatory markers. However, there are differences between aging and aging with the presence of an inflammatory stimulus, like LPS, which causes a prolonged and exaggerated immune response (Xie et al., 2003). Microglia and astrocytes of aged mice are prone to exhibit an uncontrolled LPS-induced inflammatory response (Nava Catorce and Gevorkian, 2016). Thus, the combination of aging and an inflammatory stimulus can cause an overdrive of normal physiological aging mechanisms, leading to severe anatomical degeneration in the CNS and subsequent neurological deficits (Rosczyk et al., 2008; Norden and Godbout, 2013; Wong, 2013).

Cholinergic neurons in the BF show a lower excitability profile compared to their neighboring non-cholinergic neurons, and their basic properties do not significantly change between adolescence and young adulthood in mice (López-Hernández et al., 2017). However, older animals have a lower excitability profile in cholinergic neurons than younger animals, and there is an age-dependent bi-phasic profile of cholinergic neurons during physiological aging, whereby excitability increases in adult mice (9–12 months old), but excitability decreases in aged mice (>18 months; Kékesi et al., 2019). Together, this suggests age-dependent susceptibility of BF neurons to changes in excitability. Interestingly, the number of cholinergic neurons also decreases with age in humans (Schliebs and Arendt, 2011).

Some research groups have assessed the susceptibility of cholinergic neurons to neuroinflammation throughout aging. In these studies, LPS injections were used to induce glial cell activation, which leads to a release of pro-inflammatory cytokines and ROS (Goossens et al., 1995; Minghetti and Levi, 1995). A study on 3 months old rats observed a significant loss of cholinergic neurons and extensive inflammation, including astrocyte and microglia activation, after direct BF LPS injections (Wenk et al., 2000). LPS can also be administrated *via* peripheral injections to induce a milder inflammatory response in the brain and allow for the investigation of glial activation at different ages. Kékesi et al. (2019) noticed that LPS peripheral injections slightly affect electrophysiological properties of cholinergic neurons in younger mice (3–6 months), but drive hyperexcitability in older mice (9–12 months). Surprisingly, they did not report LPS-induced excitability differences in aged mice (>18 months). The mechanism underlying the altered excitability of cholinergic neurons and neuroinflammation is yet to be resolved, although the authors proposed a connection between the bi-phasic pattern and LPS injections. The pattern is abolished due to dysregulation of calcium homeostasis and changes in membrane resistance of cholinergic neurons, which is caused by glial activation subsequent LPS-induced neuroinflammation (Kékesi et al., 2019).

Neurodegenerative diseases including Alzheimer’s and Lewy Body Dementia (LBD) share two characteristics in common: a prevalence that increases with age and is associated with elevated neuroinflammatory markers. This relationship has led to the hypothesis that pathogenic mechanisms underlying age-related neurodegenerative disease involve changes in the CNS cells responsible for the immune response during aging (Wong, 2013).

#### Alzheimer’s Disease

AD is the most common cause of age-related dementia, and its pathological hallmarks include senile plaques and neurofibrillary tangles along with extensive neurodegeneration, characterized by progressive cognitive impairment (Francis et al., 1999). Several environmental and genetic factors increase the risk for AD development (Armstrong, 2019). Aging is one of the strongest risk factors for AD, and mechanisms for this include an age-related increase in microglial activation (Schuitemaker et al., 2012), degradation of the blood-brain barrier, and a decrease of glucose influx (Mooradian, 1988; Mooradian et al., 1991; Shah and Mooradian, 1997). Other risk factors associated with AD include several genes associated with the formation of Aβ aggregates or tau neurofibrils, as well as genes involved in the modulation of the immune system (Giri et al., 2016). Some environmental factors are also associated with AD include stress (Baglietto-Vargas et al., 2015; Caruso et al., 2018, 2019) or systemic inflammation (Holmes et al., 2009; Holmes, 2013; Takeda et al., 2014; Lim et al., 2015). One hypothesis suggests that in AD (and other neurodegenerative diseases), microglia can be “primed” which later leads to a disproportionated response towards a pro-inflammatory stimulus (Dilger and Johnson, 2008; Lue et al., 2010; Cunningham, 2013; Niraula et al., 2017; Sfera et al., 2018). Several factors can induce this priming. During systemic inflammation, pro-inflammatory cytokines are expressed in response to a pathogen invasion or tissue injury, and they can reach the CNS. This can prime microglia which, combined with other risk factors e.g., genetic mutations associated with AD, leads to chronic neuroinflammation and neurodegeneration. Microglia can also be primed by exposure to Aβ aggregates or other abnormal proteins, to glucocorticoids, or by the loss of their inhibition because of a genetic predisposition (which could also be favorited by aging). Subsequent systemic inflammation then leads to chronic neuroinflammation and neurodegeneration (Cunningham, 2013). It is relevant to mention that the vagus nerve, which uses ACh as the main neurotransmitter, is involved in the regulation of the innate immune response during systemic inflammation. The vagus nerve is thought to be an important component for the bidirectional exchange between the CNS and the peripheral immune system (Thayer and Sternberg, 2010; Kox and Pickkers, 2015) and is involved in the cholinergic anti-inflammatory pathway (Zila et al., 2017).

##### Alzheimer’s Disease and the Basal Forebrain Cholinergic System

The early establishment of the “cholinergic hypothesis” of AD was based on deficits in cholinergic function in AD (Bartus et al., 1982; Francis et al., 1999; Hampel et al., 2019). Indeed, interest in the cholinergic hypothesis of AD has attracted recent attention, as virtually all older adults over the age of 70 show progressive accumulation of misfolded extracellular amyloid and intracellular over-phosphorylated tau proteins, but progressive neurodegeneration that accounts for AD-related cognitive impairment occurs only in ~10% of this population (Kok et al., 2009; Deture and Dickson, 2019). These findings suggest that “proteinopathies” alone are not a sufficient driving force to lead to progressive neurodegeneration and cognitive loss. Recent evidence indicates microglia may play a key role in neurodegenerative processes. Indeed, microglial Aβ phagocytosis is identified as one of the mechanisms to reduce Aβ burden and has been proposed as a therapeutic target for AD (Bard et al., 2000). As opposed to the “amyloid hypothesis” to explain AD etiology, the “inverse Warburg hypothesis” is a theory centered on metabolic mechanisms and more particularly on the cooperation between neurons and astrocytes, the latter playing a fundamental role in energy supply and glutamate-glutamine cycle, that could have critical implications for neurodegeneration (Bélanger et al., 2011; Demetrius et al., 2015). Thus, astrogliosis might have major metabolic consequences on neurons.

Cholinergic neurons are amongst the most energy-consuming neurons as they require the production of supplementary acetyl-CoA by mitochondria which makes them highly sensitive to toxicity from excess microglial activation (Wenk et al., 2000) and oxidative stress (Wurtman, 1992; Fass et al., 2000; McKinney, 2005; Szutowicz et al., 2013). These neurons have also been shown to be vulnerable to the activation of the hypothalamic-pituitary-adrenal axis early in life (Aisa et al., 2009). As mentioned earlier, the central cholinergic system is involved in cognitive processes such as executive function, attention, and memory (Levin and Simon, 1998; Ballinger et al., 2016; Prado et al., 2017; Solari and Hangya, 2018) but also in the regulation of the vascularization of the brain (Sato and Sato, 1995; Van Beek and Claassen, 2011). In AD, amongst the two main cholinergic nuclei of the brain, the cholinergic neurons of the BF appear to be the ones to degenerate compared to the cholinergic neurons of the BS which seem to be relatively spared (Woolf et al., 1989; Lehéricy et al., 1993; Kotagal et al., 2012; Schmitz and Nathan Spreng, 2016; Schmitz et al., 2018). Nonetheless, cholinergic neurons of the BS are still affected and exhibit neurofibrillary tangles or/and amyloid plaques (Parvizi et al., 2001). Interestingly, the cholinergic neurons of the BS are involved in ocular saccades (Kobayashi and Isa, 2002). This may raise the question of a link between impairment of the BS cholinergic neurons and its involvement in visual complaints of some AD patients, which is sometimes their primary reason for a consult (Kobayashi and Isa, 2002). The degeneration of BF cholinergic neurons appears to coincide with cognitive decline exhibited by AD patients (Grothe et al., 2014; Ballinger et al., 2016; Schmitz and Nathan Spreng, 2016). Thus, the loss of cholinergic inputs to the hippocampus from the BF could contribute to AD memory impairment, as the hippocampus is a key structure in learning and memory (Bartsch and Wulff, 2015). The loss of cholinergic input to the frontal cortex also affects executive function and cognitive flexibility (Prado et al., 2017), which also impairs memory as patients can no longer develop efficient memorization strategies. In AD patients, there is a hypometabolism of the temporoparietal area, the frontal cortex, and the posterior cingulate cortex (Mosconi, 2005; McMurtray et al., 2008). This hypometabolism is likely due to the loss of cholinergic inputs from the BF, which in turn leads to functional impairment in downstream temporoparietal regions (Grothe et al., 2016). This may coincide with hypoperfusion due to the death of cholinergic neurons, as the BF is involved in brain blood flow regulation (Sarter and Bruno, 2004; Van Beek and Claassen, 2011). In return, this hypoperfusion could lead to the death of even more cholinergic neurons as the cholinergic neurons are energy-deprived. In rats, cholinergic inputs from the BF are responsible for increasing cerebral blood flow in the hippocampus (Nishimura et al., 1992; Sato et al., 2004); this can be impaired by limited NO production *via* a decrease of neuronal NO-synthase catalytic activity (Hartlage-Rübsamen and Schliebs, 2001). In humans, it has been shown that greater blood flow into the hippocampus is correlated with a better performance in spatial memory in older adults (Heo et al., 2010) and a greater hippocampal vascular reserve might be a protective factor against cognitive loss (Perosa et al., 2020).

**Figure 1:**
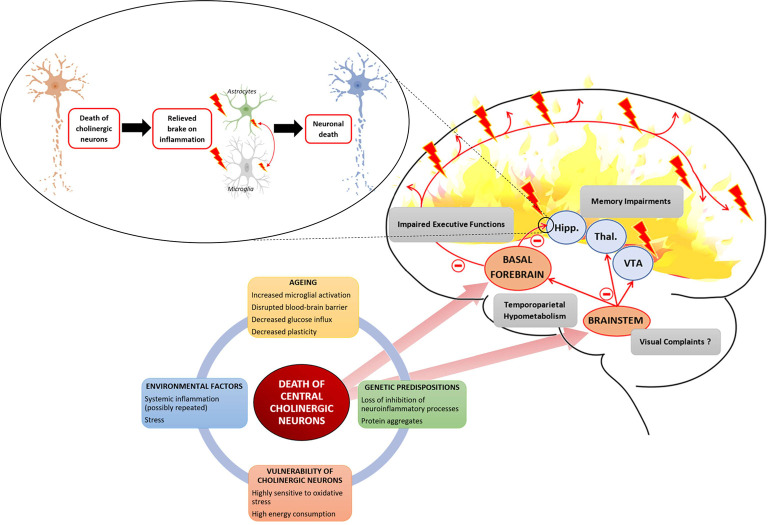
Several interacting factors can lead to the death of central cholinergic neurons. Aging with an increased microglial activation disrupted the blood-brain barrier and decreased neuronal plasticity provides a breeding ground to which environmental factors, such as stress or systemic inflammation, and genetic factors, such as loss of inhibition of neuroinflammatory process or abnormal protein leading to aggregates, are added. Cholinergic neurons are highly demanding in energy and highly sensitive to oxidative stress. The two main cholinergic populations of the central nervous system (CNS) are located to the brainstem (BS) and the basal forebrain (BF). The cholinergic neurons of the BF are projecting to the hippocampus (Hipp.) and throughout the neocortex. The cholinergic neurons of the BS are projecting to the thalamus (Thal.), the ventral tegmental area (VTA), and the BF. Death of BF cholinergic cells leads to a loss of acetylcholine (Ach) influx in the hippocampus which is involved in memory impairment but also relieves a brake on neuroinflammation. The loss of cholinergic input to the PFC leads to impaired executive function. Concerning cholinergic death in the BS, it raises the question of its association with the inaugural visual complaints expressed by Alzheimer patients as it controls ocular saccades. Finally, the death of central cholinergic neurons is associated with a temporoparietal hypometabolism.

The relationship between central cholinergic degeneration and AD proposed in this review is summarized in Figure 1.

As mentioned earlier, microglia can function by displaying persistent chronic inflammation which can lead to neurodegeneration (Zamanian et al., 2012; Sochocka et al., 2017; Tohidpour et al., 2017). One of the mechanisms involved in the establishment of neurodegeneration is increased phagocytosis and upregulation of an innate immune receptor protein on the cell membrane, TREM2, which aids in the phagocytosis of accumulated amyloid and tau debris (Klinkenberg et al., 2011). During phagocytosis of the accumulated debris, membrane TREM2 is released into the cerebrospinal fluid (CSF) as soluble (s)TREM2, with little apparent inflammation (Ewers et al., 2019; Parhizkar et al., 2019). This process may be neuroprotective during the earliest stages of amyloid pathology, but with the subsequent occurrence of tau pathology associated with a larger increase in CSF sTREM2, may reflect the transition of microglia to a detrimental phenotype (Leyns et al., 2017; Schmitz et al., 2020). Activated microglia upregulate multiple proinflammatory cytokines in response to neuronal amyloid and tau accumulation (Sarlus and Heneka, 2017). Activated microglia are also linked to the proliferation of neurotoxic reactive astrocytes *via* the complement component C3, which is the activating gene of the complement cascade, highly upregulated in the cross-talk between activated microglia and reactive astrocytes (Liddelow et al., 2017). Dysregulation of cholinergic modulation, possibly due to early age-related BF cholinergic neuronal loss, may remove an important check on the proliferation of pro-inflammatory activated microglia. Indeed, the loss of cholinergic inputs to the hippocampus from the BF might favor neuroinflammation in the hippocampus as the anti-inflammatory effect of the ACh is impaired. This view was supported by a recent study by Schmitz et al. (2020) which shows that an increased magnitude of BF degeneration in preclinical adults was associated with abnormally elevated levels of sTREM2 in the CSF and increased levels of peripheral blood C3. The authors hypothesized that damage to cholinergic neurons in the aging brain might result from a disruption to lipid metabolism, possibly due to an apolipoprotein E4 (APOE4) genetic background (Schmitz et al., 2020). Interestingly, they showed that increased BF degeneration and C3 expression were most pronounced in preclinical APOE ɛ4 carriers, despite equivalent levels of CSF Aβ and pTau (Schmitz et al., 2020). Previous studies validate these results, as APOE4 glia secrete less lapidated cholesterol and fatty acids which are unable to be transported into neurons, and deprived neurons of energy to build, repair, and maintain synapses and axons (Kanekiyo et al., 2014; Hu et al., 2015). The larger size of the cholinergic axon arbors presents biological challenges such as a large expenditure of resources for growth, maintenance, repair, and axonal transport, making these cells susceptible to age-related changes (Wu H. et al., 2014). Overall, the data presented suggest that the loss of afferent cholinergic BF input in AD may disrupt anti-inflammatory cholinergic signaling and exacerbate microglial proinflammatory responses in the presence of other age-related “proteinopathies,” leading to further neurodegeneration and cognitive loss in the later stages of the disease.

##### Reduced Cholinergic Transmission and the Therapeutic Effect of α7nAChR in AD

Decreased expression of nicotinic receptors can be found in the brain of AD patients. This decrease is not uniform depending on the sub-unit or the brain area considered. In a study using human brain homogenates, a significant decrease in protein levels of α7nAChR (about 17%) and α4nAChR (about 40%) was found in the superior temporal gyrus in AD patients compared to controls (Burghaus et al., 2000). Similarly, a different study detected a decrease in protein levels of 35% for the α4 subunit, 25% for the α3 subunit, and 36% α7 subunit was found in the hippocampus, and in the temporal cortex, there was a decrease of 47% for the α4 subunit and 29% for the α3 subunit, with no change for the α7 subunit (Guan et al., 2000). There was no change in the β2 subunit in these areas (Guan et al., 2000). When looking specifically at the expression of α7nAChR in the astrocytes, Teaktong et al. (2003) found more α7-immunoreactive astrocytes in the hippocampus and entorhinal cortex of AD patients compared to controls. Also, in human post mortem AD brain tissue, microglia accumulates on Aβ deposits and expressed α7nAChRs (Takata et al., 2010). Together, this suggests a reduction in glial α7nAChR is evident in several brain regions in AD.

Preclinical data suggest that increasing the expression and function of glial α7nAChR may be protective in AD. In primary cultured rat microglia, the positive modulation of nAChRs by galantamine or stimulation of nAChRs by nicotine increases Aβ phagocytosis (Takata et al., 2010). Moreover, these changes were closely associated with reduced Aβ burden in the brain and enhancement of memory in the APdE9 transgenic mouse model of AD (Takata et al., 2010). A recent study published by the same group confirmed the subtype of nAChR involved in these beneficial effects as α7, and α7nAChR selective agonist 3-[(2, 4-dimethoxy)benzylidene]-anabaseine dihydrochloride (DMXBA) promoted Aβ phagocytosis in primary cultures of rat microglia (Takata et al., 2018). They also reported the administration of DMXBA to ApdE9 mice attenuated brain Aβ burden and memory dysfunction (Takata et al., 2018). Moreover, DMXBA also repressed γ-secretase activity in human neuroblastoma cells and in transgenic mouse brains (Takata et al., 2018). In another study, long-term treatment of aged 3xTg-AD mice with the selective α7nAChR agonist, A-582941 (12 mg/kg/day, for 3 months, from 15 to 18 months of age), completely restored cognition in 3xTg-AD mice to the level of that in age-matched non-transgenic controls (Medeiros et al., 2014). Overall, this suggests that selective activation of microglial and neuronal α7nAChRs promotes Aβ phagocytosis and suppresses neuronal γ-secretase activity respectively, lessening Aβ burden and cognitive impairment.

Stimulation of α7nAChRs on microglia and neurons ameliorates brain Aβ burden and cognitive impairment *via* two distinct mechanisms (Takata et al., 2010, 2018). In microglia, stimulation of α7nAChR increases cytosolic Ca^2+^ and consecutively activates calcium-dependent pathways for actin reorganization through CaM-CaMKII (Ca^2+^/calmodulin-dependent protein kinase II) and CaM-Rac1-WAVE (CaM- Ras-related C3 botulinum toxin substrate 1-Wiskott-Aldrich syndrome protein family verprolin-homologous protein) signaling pathways and subsequently may promote microglial Aβ phagocytosis. In neurons, α 7nAChR stimulation may downregulate presenilin 1 (PS1) expression and subsequent suppression of γ-secretase activity.

One of the proposed mechanisms explaining Aβ-related neurotoxicity is microglia-related oxidative stress and neuroinflammation *via* nicotinamide adenine dinucleotide phosphate (NADPH) activation (Kim et al., 2007). The neuroprotective function of nicotine may be mediated by suppressing Aβ-induced ROS production in microglia. Nicotine suppresses ATP release and thus inhibits NADPH oxidase activation, completely blocking Ca^2+^ influx in Aβ-stimulated microglia (Della Bianca et al., 1999; Moon et al., 2008). Taken together, these findings suggest that reduced expression or inactivation of microglial and neuronal α7nAChRs receptors may contribute to some pathological changes and cognitive impairment observed in AD.

A therapeutic approach that targets α7nAChRs is already being explored by pharmaceutical companies not only for AD but also for disorders such as PD, schizophrenia, or attention deficit hyperactivity disorder (Yang et al., 2017).

#### Lewy Body Dementia

Neurodegenerative diseases characterized by the presence of intraneuronal Lewy bodies (constituted primarily of α- synuclein) and Lewy neurites are termed Lewy body diseases and are considered the hallmark of dementia with Lewy bodies and PD (Duda, 2004). The principal Lewy body diseases are PD, Parkinson’s disease dementia, and LBD (Barrett et al., 2020). LBD is recognized as a common cause of cognitive deterioration in the elderly and is the second most common cause of neurodegenerative dementia after AD (Tiraboschi et al., 2002; Ferman and Boeve, 2007). It is well-established that cholinergic input from the nucleus basalis of Meynert in the BF is required for cognitive function, and degeneration of the nucleus basalis of Meynert in LBD contributes to the weakening of cognitive function observed in LBD patients. LBD is also characterized by neuropsychiatric symptoms such as psychosis with visual hallucinations and depressive symptoms associated with BF cholinergic degeneration. More compelling evidence of cholinergic BF degeneration in LBD comes from cholinesterase inhibitor trials (Barrett et al., 2020). One such early clinical trial using rivastigmine, a cholinesterase inhibitor, improved hallucinations, delusions, apathy, and anxiety in patients with LBD (Mckeith et al., 2000). In LBD, ACh downregulation is generally thought to be more severe than that in AD (Kitajima et al., 2015) and autoradiographic studies on postmortem brains showed reduced nAChR in the cortex and hippocampus in PD (Rinne et al., 1991; Lange et al., 1993; Barrett et al., 2020). Rinne et al. (1991) showed that a reduction in nicotinic receptor binding in the frontal cortex correlated with the severity of dementia in PD and AD (Rinne et al., 1991). While there was a reduction in both α7 and non-α7nAChRs in LBD, only the decrease in α7 receptors correlated with cholinergic denervation as measured by ChAT activity (Reid et al., 2000). Moreover, Court et al. (2001) reported a loss of α7nAChRs in the temporal cortex of dementia with Lewy Bodies (DLB) patients associated with visual hallucinations and delusional misidentification (Court et al., 2001). Together, this indicates a role for cortical and hippocampal nAChRs, and in particular α7nAChRs in LBD, which may be linked to behaviors such as hallucinations, delusions, apathy, and anxiety in LBD patients. All the above-mentioned studies focused on α7nAChRs as a whole, without differentiating them into neuronal or glial subtypes. However, direct evidence of inflammation in LBD is growing, with augmented microglial activation identified at post-mortem (Togo et al., 2001), as well as a recent study reporting microglial activation in key brain regions associated with disease pathology (Surendranathan et al., 2018). Immunohistochemical studies carried out on post-mortem brains of AD and LBD patients reported an increase in the number of astrocytes double-labeled with α7nAChR and GFAP antibodies, in most areas of the hippocampus and entorhinal cortex in AD, but not in LBD (Teaktong et al., 2003, 2004). Thus, further pre-clinical and clinical evidence is required to support the direct involvement of glial α7nAChRs in LBD to establish an association with neurodegenerative pathology in this disease.

#### Parkinson’s Disease

PD is a common neurodegenerative disease in the elderly and is pathologically characterized by the loss of dopaminergic neurons in the substantia nigra (SN). However, the origin of PD and mechanisms of neuronal degeneration are not fully understood. Compelling evidence indicates that CNS glia has an initiating role in PD pathophysiology. While PD patients show significant loss of dopaminergic neurons in the SN, the degeneration of the BF cholinergic system is a factor in dementia associated with PD (Maurer and Williams, 2017). There is some evidence for the contribution of the BF function to dementia in PD. Administration of the neurotoxin 1-methyl-4-phenyl-1,2,3,6-tetrahydropyridine (MPTP) is used to model PD in rodents. A metabolite of MPTP in astrocytes, 1-methyl-4-phenylpyridinium ion (MPP+), can block mitochondrial complex I, leading to selective dopaminergic neurodegeneration, similar to that present in PD (Jakowec and Petzinger, 2004). LPS administration is also used to model PD in animals, as it activates glial cells and induces inflammatory changes and dopaminergic neurodegeneration (Dutta et al., 2008). In a study using *in vivo* and *in vitro* murine models of PD, microglia and astrocytes in the SN increased in number and displayed more activation-associated morphology compared to controls, due to neuroinflammation triggered by MPTP/MPP+ or LPS (Liu et al., 2012). Systemic administration of nicotine alleviated MPTP-induced perturbed behavioral symptoms (by improving motor coordination) and protected against dopaminergic neuron loss, as well as microglia and astrocyte activation in the SN (Liu et al., 2012). Protective effects of nicotine were abolished by the administration of the α7nAChR-selective antagonist methyllycaconitine (MLA), indicating α7nAChRs mediate neuroinflammation and PD-like behaviors in these models (Liu et al., 2012). Interestingly, in primary cultured mouse microglia and astrocytes, pre-treatment with nicotine suppressed MPP+-induced or LPS-induced glial activation and number, evidenced by decreased production of TNF-α and inhibition of extracellular regulated kinase1/2 (Erk1/2) and p38 activation in glia (Liu et al., 2012). These effects were also reversed by MLA, suggesting a role for microglial and astrocyte α7nAChR in inflammatory responses relevant to PD. A recent study assessed the anti-apoptotic effects of an α7nAChR agonist PNU-282987 in primary cultured astrocytes treated with MPP+ (Hua et al., 2019). They showed that PNU-282987 promoted the viability of astrocytes, alleviated MPP+ induced apoptosis by upregulating the expression of the antiapoptotic protein Bcl-2, and downregulated the expression of the apoptotic protein Bax and cleaved-caspase-3 (Hua et al., 2019). This suggests that PNU-282987 may be a potential drug for restoring astroglial function in the treatment of PD *via* astroglial α7nAChR-JNK-p53 signaling.

Recent literature has described how the vagus nerve may play an important role in the pathogenesis of PD. Reflecting our previous discussion on the function of the vagus in the CAP, disruption in vagal cholinergic signaling could promote a neuroinflammatory state. Studies using murine models have shown that in advanced age, vagal visceral sensory innervation of the gastrointestinal tract is reduced (Phillips and Powley, 2001; West et al., 2019). Additionally, in aging the vagus nerve is found to reduce in size (Walter et al., 2018) and afferent fibers are found to regress and undergo dystrophic changes (Phillips et al., 2010). Further research is required to understand how changes to vagal afferent fibers might impact neuroimmune signaling and the CAP. Recent literature discussing the vagus nerve during aging focuses on its role in the gut-brain axis and PD. It has been suggested that truncal vagotomy could reduce the risk of PD development (Liu et al., 2017). Prodromal gut dysfunction and inflammation have been found to precede motor symptoms of PD by decades (Nair et al., 2018). The vagus nerve acts as the main link between the gut-brain axis and inflammation or a prion-like cascade of α-synuclein may be propagated to the CNS *via* the vagus nerve (Liddle, 2018). Even throughout life, gut infections by vagal signaling could influence an endophenotype of microglial cells that promotes neurodegeneration in later decades (Desplats et al., 2019). A better understanding of vagal neuroimmune signaling could provide insight into the progression of PD.

#### Dementias Associated With Chronic Pain

Chronic pain occurs due to altered neuronal plasticity, and this includes altered sensitization of peripheral primary sensory neurons in the dorsal root ganglia and trigeminal ganglia, as well as the sensitization of central nociceptive neurons in the spinal cord, trigeminal nucleus, BS, and cortex (Ji et al., 2014). Clinically, chronic pain is defined as pain lasting more than 3 months and is typically characterized by hyperalgesia (an increased response to noxious thermal and mechanical stimuli) and allodynia (nociceptive responses to normally innocuous stimuli such as light touch; Ji et al., 2014).

Neuroinflammation plays an important role in the induction and maintenance of chronic pain (Ji et al., 2014). Unlike acute inflammation that produces transient central sensitization, chronic pain is associated with producing a long-lasting or permanent central sensitization that persists even after the acute inflammation has been resolved (Ji et al., 2014). Chronic pain affects up to 30% of older adults worldwide (Larsson et al., 2017). Generally, it is associated with abnormalities in sensory processes, but it is highly associated with cognitive, emotional, and social dysfunction (Malfliet et al., 2017). The Einstein Aging Study showed that chronic pain is associated with dementia, and 10% of the 1114 elderly participants in the study developed dementia over 4.4 years (Katz et al., 2012; Ezzati et al., 2019). Moreover, a recent review summarized evidence for a possible mechanism of chronic pain-induced AD pathogenesis through LC-noradrenaline (NE) system dysfunction and microglial neuroinflammation (Cao M. et al., 2019). They suggested that chronic pain may induce pathological activation of the LC-NE system and lead to an increased NE release in brain areas such as the PFC and hippocampus, which could then result in chronic pain-induced microglial pro-inflammatory activation. Thus, this microglial pro-inflammatory activation may aggravate AD pathogenesis *via* decreased Aβ phagocytosis, increased tau seeding, loss of synaptic function, and cytokine-induced neuron death in these brain regions (Cao S. et al., 2019).

While the hippocampus appears to play a vital role in pain perception and processing, this limbic structure shows high expression of microglial cells (Soleimannejad et al., 2007; Tan et al., 2020). As mentioned previously, while microglia contribute to the early development of chronic pain, dysregulation of astrocyte function is required to sustain developed chronic pain (Jha et al., 2012). Furthermore, hippocampal ACh is an important mediator in regulating chronic pain-induced behavioral deficits and studies have demonstrated antinociceptive effects of cholinergic compounds (Jiao et al., 2009; Chen et al., 2016). The compound 3a,4,5,9b-Tetrahydro-4-(1-naphthalenyl)-3H-cyclopentan[c]quinoline-8-sulfonamide (TQS) is a α7nAChR PAM. A study using a mouse model of LPS-induced neuroinflammatory pain demonstrated that TQS reduced hippocampal microglial activation and hyperalgesia and allodynia, an effect that was reversed by pre-treatment with the α7nAChR antagonist MLA (Abbas and Rahman, 2016). Microglia regulate the transcription of various pain mediating genes, including the inhibitor of κB (IκB) mRNA (Abbas et al., 2017). Within the cytoplasm of resting-state microglia, IκB is bound to NF-κB, preventing inflammatory signaling (Abbas et al., 2017). When stimulated, IκB becomes phosphorylated and unmasks NF-κB from an inactive to an active state (Abbas et al., 2017). Activated NF-κB then translocates to the nucleus and positively regulates the transcription of various pain mediating genes (Li and Verma, 2002; Abbas et al., 2017). Increased IκB mRNA and CD11b mRNA, which is a microglial activation marker in the brain, are expressed simultaneously during hyperalgesia and allodynia (Loram et al., 2010; Abbas et al., 2017). Recently, Abbas and Rahman, demonstrated the α7nAChR PAM decreases IκB and CD11b gene expression and microglial activation associated with hyperalgesia and allodynia, by targeting microglial α7nAChR in the hippocampus (Abbas et al., 2017). Thus, targeting excessive neuroinflammation could offer new therapeutic approaches when managing chronic pain related neurological and cognitive disorders. In addition, these findings suggest that a α7nAChR PAM may represent a new treatment approach for allodynia and hyperalgesia associated with microglial activation during chronic pain (Abbas and Rahman, 2016; Abbas et al., 2017).

## Conclusions

This review has summarized research on the cholinergic modulation on glial function, with a particular focus upon aging and chronic neuroinflammation in the CNS. The BF provides cholinergic inputs into various brain regions including the hippocampus and the cortical areas, regions with important higher cognitive functions. It has been shown that nicotinic receptor α7nAChR is expressed on hippocampal neurons, and is involved in neuroprotective processes. This is evidenced by the direct activation of neuronal α7nAChR reversing cholinergic pathway-dependent cognitive deficits. The nicotinic BF inputs to hippocampal regions not only modulate neuronal α7nAChR receptors but also have a substantial influence upon hippocampal glia. Both astrocytes and microglia are involved in the cholinergic anti-inflammatory pathway *via* α7nAChR expression. This has been demonstrated by modulation of microglial and astrocytic nicotinic receptor activity, which results in Aβ phagocytosis and cognitive improvement in neurodegenerative disease states. Expression of GDNF is stimulated by the activation of α7nAChRs which inhibit microglial activation, leading to neuronal protection. Wider and more extensive studies on microglial α7nAChR may lead to the use of microglial function in the diagnostic screening of dementias.

The effects of the cholinergic BF projections on anti-inflammatory and anti-oxidant processes is not a simple unilateral interaction between BF projections and neurons, microglia, and astrocytes, but a multilateral integration of these components. This is evident in the extensive cellular relationship between microglia and astrocytes. Hence, the disruption of the cholinergic BF system, *via* neurodegeneration, energy loss, infection, or aging, may play a significant role in the development of neurodegenerative diseases.

## Author Contributions

All authors have contributed to conceiving the idea of this review, reviewed the literature, and contributed to the writing and editing of this manuscript. All authors have approved this manuscript. RG and IW have contributed equally to this manuscript as the first authors. All authors contributed to the article and approved the submitted version.

## Conflict of Interest

The authors declare that the research was conducted in the absence of any commercial or financial relationships that could be construed as a potential conflict of interest.

## Abbreviations

ACh: acetylcholine
AChE: acetylcholine esterase
AD: Alzheimer’s disease
ANS: autonomic nervous system
APOE4: apolipoprotein E4
ATP: adenosine tri-phosphate
Aβ: Amyloid β
BBB: the blood-brain barrier
BDNF: brain-derived neurotrophic factor
BF: basal forebrain
BS: Brainstem
CCR: CC chemokine receptor
ChAT: choline acetyltransferase
CNS: central nervous system
COX-2: cyclooxygenase-2
CSF: cerebrospinal fluid
CXCL: CXC motif ligand
DMXBA: 3-[(2,4-dimethoxy)benzylidene]-anabaseine dihydrochloride
GABA: γ-aminobutyric acid
GDNF: glial cell line-derived neurotrophic factor
GFAP: glial fibrillary acidic protein
HO1: heme oxygenase
IL: interleukin
INF: interferon
IP: inducible protein
IP3: inositol-3-phosphate
IκB: an inhibitor of nuclear factor κB
JAK: Janus kinase
JNK: c-jun N-terminal kinase
LBD: Lewy body dementia
LPS: lipopolysaccharide
mAChR: muscarinic acetylcholine receptor
MAPK: mitogen-activated protein kinase
MHC: major histocompatibility complex
MLA: methyllycaconitine
MPTP: 1-methyl-4-phenyl-1,2,3,6-tetrahydropyridine
nAChR: nicotinic acetylcholine receptor
NADPH: nicotinamide adenine dinucleotide phosphate
NF-κB: nuclear factor-κB
NO: nitric oxide
Nrf2: nuclear factor erythroid 2-related factor 2
ORM2: orosomucoid 2
PAM: positive allosteric modulator
PD: Parkinson’s disease
PFC: the pre-frontal cortex
PI3K: phosphatidylinositol-3-kinase
PLC: Phospholipase C
PNS: peripheral nervous system
ROS: reactive oxygen species
STAT: signal transducer and activator of transcription
TGF: transforming growth factor
TNF: tumor necrosis factor
TQS: 3a,4,5,9b-Tetrahydro-4-(1-naphthalenyl)-3H-cyclopentan[c]quinoline-8-sulfonamide
VTA: a ventral tegmental area
α7nAChR: α7 nicotinic acetylcholine receptor.
